# Novel Load Systems for *In Vitro* Testing of Biomaterials and Medical Devices

**DOI:** 10.3390/ma16020465

**Published:** 2023-01-04

**Authors:** Cristina Bignardi, Mara Terzini

**Affiliations:** 1 Department of Mechanical and Aerospace Engineering, Politecnico di Torino, 10129 Turin, Italy; 2 Polito^BIO^Med Lab, Politecnico di Torino, 10129 Turin, Italy

In the mechanical characterization of materials or devices, the real load conditions to which they will be subjected in their operational environment must often be simulated by starting from the availability of universal testing machines. Although sophisticated, these machines only apply uniaxial loads or, at most, simultaneously apply loads along few degrees of freedom.

With the aim of investigating the structural behavior of materials and devices, usually researchers check the availability of adequate existing testing machines before inventing new loading systems and verify the existence of indications in the International Standards or in the literature. In the field of biomedical engineering complexity grows due to the peculiar loads (i.e., the physiological ones) and environments to which biomaterials and medical devices are subjected. They, indeed, will be coupled to the receiving organs or tissues once implanted.

This Special Issue was born from the desire to give space to the imagination of the researchers involved in the conception and design of experimental benches able to simulate the real load conditions under which biomaterials and medical devices will operate. In the Special Issue, seven papers were published which explore different contexts in which the design of novel loading systems constituted the originality of the research. They range from the characterization of the mechanical behavior of biomaterials [1,2] and of biological tissues [3], to the verification of the structural behavior of more or less complex medical devices [4,5], from the design of tests for verifying the stability of articular prosthetic components once coupled to the bone [6], to the simulation of the erosive effect of surgical meshes on the biological soft tissues with which they interface [7].

The aim of the investigations [1,2], in the ambit of implant fixed dental prostheses, is to determine the influence of luting agent, abutment height and taper angle on the retrievability of abutment-coping cementations in two different layouts: with one implant and with two implants connected by a bridge. The number of impulses and the impulsive force delivered during each test were recorded and used as retrievability indexes.

Only recently, ovary responsiveness to mechanical signals was exploited for reproductive purposes. Poor characterization of ovarian cortex biomechanics and of the mechanical challenge hampers reproducible and effective treatments, and prevention of tissue damages. In [3], the biomechanical response of ovarian cortical tissue from abattoir bovines is characterized for the first time. Ovarian cortical tissue fragments were subjected to uniaxial dynamic testing at frequencies up to 30 Hz, and at increasing average stresses. In [4], both mechanical and morphological properties of an innovative bandage system, useful to position and fix patients’ torsos or extremities, are reported. The creation of a new complex medical device, such as a prosthesis for amputees, requires numerous design phases accompanied by fundamental structural tests. In [5], all the design phases of a new powered ankle–foot prosthesis are reported. Sufficient primary fixation stability is the basis for the osseointegration of cementless acetabular cups. Several test methods have been established for determining the tilting moment of acetabular press-fit cups, which is a measure for their primary fixation stability. The central aim of the experimental study explained in [6] is to show the differences between the commonly used lever-out test method and the edge-load test method in which the cup insert is axially loaded during the tilting process with respect to the parameters, tilting moment, and interface stiffness. At last, the research shown in [7] deals with the phenomenon of meshes erosion whereby soft tissue becomes damaged as a result of contact with implants made from surgical mesh, a fabric-like material consisting of fibers of polypropylene or other polymers. The paper describes the design and construction of a testing machine to generate mesh erosion *in vitro*.

The Editors give special thanks to the authors and the editorial team of *Materials* for the collaborative and peer-review process. We hope that the scientific community of *Materials* will enjoy reading this Special Issue and find new ideas for future and present research works.
